# Computational identification of condition-specific miRNA targets based on gene expression profiles and sequence information

**DOI:** 10.1186/1471-2105-10-S1-S34

**Published:** 2009-01-30

**Authors:** Je-Gun Joung, Zhangjun Fei

**Affiliations:** 1 Boyce Thompson Institute for Plant Research, Cornell University, Ithaca, NY 14853, USA; 2 USDA Robert W. Holley Center for Agriculture and Health, Ithaca, NY 14853, USA

## Abstract

**Background:**

MicroRNAs (miRNAs) are small and noncoding RNAs that play important roles in various biological processes. They regulate target mRNAs post-transcriptionally through complementary base pairing. Since the changes of miRNAs affect the expression of target genes, the expression levels of target genes in specific biological processes could be different from those of non-target genes. Here we demonstrate that gene expression profiles contain useful information in separating miRNA targets from non-targets.

**Results:**

The gene expression profiles related to various developmental processes and stresses, as well as the sequences of miRNAs and mRNAs in *Arabidopsis*, were used to determine whether a given gene is a miRNA target. It is based on the model combining the support vector machine (SVM) classifier and the scoring method based on complementary base pairing between miRNAs and mRNAs. The proposed model yielded low false positive rate and retrieved condition-specific candidate targets through a genome-wide screening.

**Conclusion:**

Our approach provides a novel framework into screening target genes by considering the gene regulation of miRNAs. It can be broadly applied to identify condition-specific targets computationally by embedding information of gene expression profiles.

## Background

MicroRNAs (miRNAs) are small RNAs that play important regulatory roles in animals and plants [1]. They cause transcriptional cleavage or translational repression through binding their target mRNAs. miRNAs affect a variety of cellular processes such as development, cell proliferation, apoptosis, and stress response [2-4]. Thus identification of mRNA targets is an essential step to understand miRNA functions.

Currently several miRNA target prediction tools have been developed [1,5-10]. The majority of these algorithms are based on the sequence alignment or the minimum free energy of the hybridization. The sequence alignment or the binding energy of miRNA/mRNA pairs can sometimes hold definitive information in screening target genes. However, a number of candidate targets could be false positives due to the omission of gene expression information in the screening process.

Microarray analysis allows us to observe a number of target mRNAs down-regulated by overexpressing miRNAs [11]. Expression profiles may be useful in identifying miRNA targets that have been missed or mis-identified by the sequence analysis [12]. However, it is labor intensive to generate miRNA over-expression lines and gene expression profiles in these lines. Furthermore, it is difficult to generate gene expression profiles in diverse tissues, stages, and environments of transgenic lines due to the high cost. For these reasons, currently available gene expression profiles generated without performing the transfection experiment may also be useful sources for identifying target genes.

In this paper, we propose a novel approach for screening miRNA targets by considering gene expression profiles. Our approach is based on the model combining a machine learning tool, SVM, which uses the datasets of gene expression profiles, and a scoring method, which uses the sequences of miRNAs and mRNAs. SVM can identify unknown targets by using a kernel function that describes the similarity between given input examples. SVM was developed by Vapnik for classification of data based on statistical learning theory [13]. It has provided a number of applications in biological data analysis, including the classification of cancers, splice site identification, and the classification of protein folding [14-16]. In the present study, by employing the classifier, we first investigate whether the expression profiles in specific biological processes contain enough information for the prediction of miRNA targets. Then the properties of the combined model are analyzed and the model is applied to the genome-wide target screening.

Our method was analyzed with a validated target set, gene expression profiles and gene sequences in *Arabidopsis*. The validated target sets were collected from several literature sources that describe the experimentally verified target genes. The gene expression dataset was generated with a total of 211 conditions including different developmental series and stress treatments [17]. The ability of the SVM classifier to discriminate between target and non-target genes was analyzed using only the gene expression dataset, and then several major conditions relevant to the classification were extracted using a feature selection method. Finally, we performed the target prediction using the method combining both express profiles and sequence information. Our study suggests that gene expression profile information can be combined with other miRNA target prediction algorithms to identify targets involved in specific biological processes.

## Methods

### SVM classifier

A supervised machine-learning algorithm, support vector machine (SVM), was used to classify miRNA targets from non-targets. Recently SVM has been successfully applied to miRNA predictions as well as miRNA target predictions [18,19]. Given a kernel and a set of labeled training examples belonging to positives or negatives, SVM learns a linear decision boundary in the feature space defined by the kernel function in order to discriminate between the two classes. Then, given any unlabeled example, SVM determines whether it is positive or negative, depending on the position of its image in the feature space relative to the linear boundary. In our case, using a training set containing known verified targets and non-targets, SVM builds a model for the prediction of the test set, i.e., the unknown set. In this study we used LIBSVM, a library for support vector machines [20]. The input features of SVM are expression profiles. A training or test set is represented by $D\in {\left\{x_{i},y_{i}\right\}}_{i=1}^{N}$, *x*_*i *_= (*x*_*i*1_,..., *x*_*im*_) = and *y*_*i*_∈ {-1, 1}, where *x*_*i *_is a vector of expression ratios under different conditions from a gene *i*. If *y*_*i *_= 1, then the *i*-th gene represents a target gene, otherwise it represents a non-target gene.

### Dataset construction

A number of putative targets have been predicted from sequence analysis in previous studies. However, the predicted targets should contain a small portion of false positives. Therefore, in the present study, we used only a list of ~100 experimentally validated targets as the true positive set. Nevertheless, it is challenging to make a proper training dataset for the construction of a SVM model because of the imbalance issue in machine learning [21]: the size of the validated target set is much smaller than that of the set containing all the genes excluding the validated targets. To overcome this imbalance problem, we increased the size of the validated target set through random resampling. After we increased the size of the positive dataset by a predefined number, which we set to 1,000, we constructed the negative dataset of which the size is the same as the size of the positive data through random sampling.

### Dataset of gene expression profiles

Two expression datasets were used for miRNA target prediction. The first microarray dataset contains 79 different conditions derived from several developmental series in *Arabidopsis*. The second dataset contains 132 conditions from ten different stress treatments including light, cold, drought, genotoxic, heat, osmotic, oxidative, salt, UV-B, and wound. Affymetrix CEL files of the gene expression datasets were obtained from the Nottingham Arabidopsis Stock Centre (NASC; [22]). Both datasets were generated using the ATH1 genome array containing ~22,800 probe sets. The CEL files were processed and normalized at the probe level using the GC content based robust multi-array algorithm (GCRMA; [23]). After normalization, the average of the triplicate values was calculated for each sample. In the development dataset, the relative expression level of each gene was calculated by taking the log ratio between each expression level and the mean expression level across all the samples. The stress dataset was processed by taking the log ratio between the expression level of treatments and that of the corresponding normal cell types.

### Binding scoring between miRNA and mRNA

The most recent collection of *Arabidopsis *miRNAs in miRBase (Release 11.0; [24]) and mRNA sequences from the TAIR database [25] were obtained. Given a miRNA, the sequence alignment of the miRNA against all mRNAs was performed. The binding scoring function between miRNA and mRNA is based on the weighted summation of the numbers of mismatches, wobbles and indels described in Jones-Rhoades and Bartel [26].

### Combining gene expression profiles and binding information

**Figure 1:**
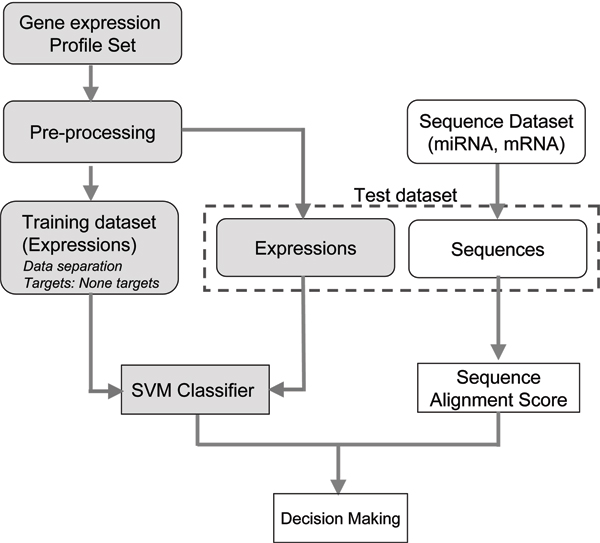
**The procedure of computational prediction of miRNA targets**. After the training dataset of gene expression files is trained by SVM, the test set is predicted by the decision making of SVM classifiers and the scoring method based on the sequence alignment.

Our target prediction strategy is based on the gene expression profiles and the binding scores between miRNA and mRNA sequences. Figure 1 shows the overall procedure of computational prediction of condition-specific miRNA targets. The prediction system consists of two parts: the SVM classifier and the binding scoring function. The expression profiles of the validated miRNA targets were used as the training dataset for modeling SVM. Then the test set is predicted by making a decision between the output of SVM and that of the scoring function. When an input gene in both outputs is indicated as a positive, it is predicted as a miRNA target.

## Results

### Classification of miRNA targets using gene expression profiles

Our prediction model classifies the targets by combining gene expression profiles and sequence information (Figure 1). Before testing the prediction model, we first investigated whether gene expression profile information can be used to discriminate the target genes from non-target genes. We applied SVM to classify target genes from non-target genes. The procedure is highlighted in gray in Figure 1. The classification is only based on patterns of gene expression between the target set and the non-target set in specific conditions. The type of SVM used is C-SVM and the type of kernel used is a linear kernel function. The gene expression dataset contains a total of 211 conditions, including 79 conditions derived from several developmental series and 132 conditions from diverse stress treatments. It has been reported that miRNAs affect the expression of a number of target genes involved in different developmental processes and stresses. We expect that both the developmental series dataset and the stress dataset are informative enough to discriminate targets from non-targets.

To achieve a good classification, it is important to define true miRNA target genes. We collected the experimentally validated miRNA targets to construct a highly accurate training dataset. The true target genes were extracted from several literature sources describing experimentally validated miRNA targets [12,27-30]. A total of 101 non-redundant target genes were collected (Additional file 1). Eighty-nine of them overlap with those in the expression dataset. 60% of these genes (53 genes) were used as the positive examples of the training dataset and the rest (36 genes) were used as the positives of test dataset. 1,000 negative examples were randomly selected from all the genes on the array excluding the validated target genes. The positive examples were increased by the number of negative examples through random re-sampling in order to keep a balance (1:1 ratio) between the size of the positive dataset and that of the negative dataset.

**Figure 2:**
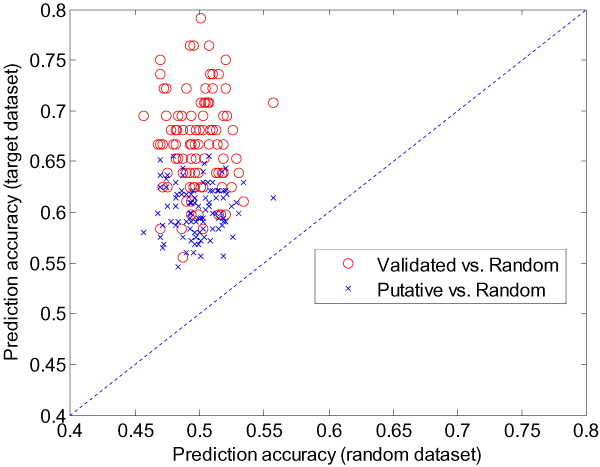
**The miRNA target prediction with SVM using the gene expression dataset**. Three datasets with different qualities, which were the validated target dataset, the putative target dataset, and the random dataset, were compared in terms of the prediction accuracy.

We investigated the prediction accuracies of using target datasets with different qualities for classification: validated, putative, and random sets (Figure 2). The validated dataset is the same as the dataset described above. The putative dataset contains 378 targets collected from several reports which were identified through computational screening [7,8,26,31], of which 328 overlapped with those in the expression dataset. The positive and training and test sets were generated using the expression profiles of these 328 putative target genes while the negative training and test sets were generated by randomly selecting genes excluding those 328 target genes. The dataset of random targets was generated by random assignment of positive or negative labels in order to observe the baseline of prediction. The size of these three datasets is identical through random re-sampling of positive examples. As expected, the target genes could be classified by prediction using only gene expression dataset. The prediction accuracy is lower when the putative target dataset is used than when the validated target dataset is used (Figure 2).

**Figure 3:**
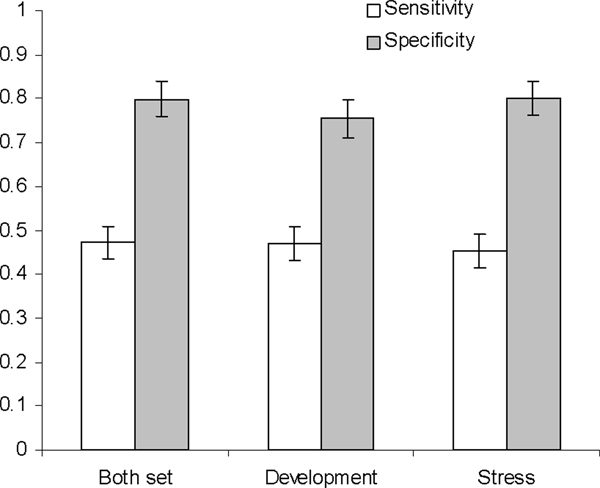
**The performance of target prediction with SVM using different gene expression sets**. The performance of target prediction with SVM using developmental- or stress-related gene expression profiles, or combined expression profiles from the two datasets.

We then performed the analysis to determine which expression datasets can be used to classify the genes more accurately. Our results indicated that no significant difference regarding the specificity and the sensitivity was found between the two datasets: the developmental dataset and the stress dataset, as well as the combined dataset (Figure 3).

We then determined which features in the expression datasets are important for the classification. The ranker search method using SVM was used to select the features. The list of the top ranked 20 features is shown in Table 1. The rank was determined by 10-fold cross validation with the training dataset, which is consisted of the validated targets (positive) and the randomly selected genes excluding the validated targets. The features from the developmental dataset and the stress dataset are highly ranked without significant disproportion, further confirming that there is no significant difference of performance between the two datasets. The full list of ranked features is shown in Additional file 2.

**Table 1:** Feature selection in the gene expression dataset.

Rank	Sample ID	Type	Tissue
1	ATGE26	D	Leaf
2	Heat-Shoots-1.0 h	S	Shoot
3	UV-B-Roots-1.0 h	S	Root
4	ATGE73	D	Pollen
5	ATGE91	D	Leaf
6	ATGE34	D	Flower
7	Drought-Roots-0.25 h	S	Root
8	Drought-Shoots-0.25 h	S	Shoot
9	UV-B-Shoots-3.0 h	S	Shoot
10	Cold-Roots-24 h	S	Root
11	ATGE97	D	Seedling
12	Drought-Roots-24.0 h	S	Root
13	UV-B-shoots-0.5 h	S	Shoot
14	ATGE55	D	Flower
15	ATGE101	D	Seedling
16	Drought-Shoots-3.0 h	S	Shoot
17	Wounding-Shoots-6.0 h	S	Shoot
18	Osmotic-Shoots-1.0 h	S	Shoot
19	Oxidative-Roots-6.0 h	S	Root
20	UV-B-Roots-6.0 h	S	Root

The top 20 ranked features for miRNA target classification. Each feature corresponds to a condition in the two expression datasets (D: developmental process and S: stress treatment).

### Classification of miRNA targets using gene expression profiles and sequence information

We then compared the efficiencies of target prediction between two different methods: the method using the combined information of expression profiles and sequence information (SVM+SC) and the method using the sequence information alone (SC). The results are shown in Table 2. SVM+SC_3 _indicates our method combining SVM classifier and SC, the scoring method based on the weighted summation of the numbers of mismatches, as well as wobbles and indels between miRNA and mRNA as described in Jones-Rhoades and Bartel [26], with 3.0 as the cutoff score. SC_1 _indicates the scoring method with a cutoff score of 1.0. TP, FP, TN and FN are the true positive, false positive, true negative, and false negative, respectively. The precision is a positive predictive value calculated by TP/(TP+FP). The sensitivity and the specificity are calculated as TP/(TP + FN) and TN/(TN + FP), respectively. The sensitivity of SVM+SC_3 _is higher than that of SC_1_, whereas its specificity is higher than that of SC_3_. Although the false positive rate of SC_1 _achieves zero, which is the same as that of SVM+SC_3_, the true positive rate is much lower. SC_3 _can predict more true positives than SVM+SC_3_, but it contains more false positives. These results suggest that the information of gene expression profiles can be utilized to increase the efficiency of miRNA target gene prediction when combined with sequence information.

**Table 2:** Comparison of predictions using different methods.

	SVM+SC_3_	SC_1_	SC_3_
TP (True Positive) rate	0.36	0.20	0.83
FP (False Positive) rate	0.00	0.00	0.03
TN (True Negative) rate	1.00	1.00	0.97
FN (False Negative) rate	0.64	0.80	0.17
Sensitivity (TP/(TP+FN))	0.36	0.20	0.83
Specificity (TN/(TN+FP))	1.00	1.00	0.97
Precision (TP/(TP+FP))	1.00	1.00	0.97

SVM+SC_3_, the method combining the SVM classifier and the scoring method based on the sequence matches. SC_τ _indicates the score cutoff, *τ*. The results were obtained with 100 test sets.

### Genome-wide identification of miRNA target genes associated with developmental processes and stress responses

We extracted the target genes identified by our classifier (SVM+SC_3_) excluding those that have been validated in *Arabidopsis*. The training dataset was generated as described in the previous section. Since the classification is dependent on the expression dataset, these targets may be involved in the corresponding biological process. The top 20 ranked genes predicted as the development-related and stress-related targets are listed in Tables 3 and 4, respectively. A number of genes retrieved by the classifier have reported roles in the corresponding developmental processes and stress responses, while the functions of most targets we identified are not clear.

**Table 3:** Top 20 target genes associated with the developmental series.

Locus ID	miRNA	Rate	Description
At1g69440*	miR854	0.97	Encodes ARGONAUTE7
At1g62930	miR400	0.83	Similar to pentatricopeptide (PPR) repeat-containing protein
At5g47250	miR472	0.82	Disease resistance protein
At3g15270*	miR156	0.78	Squamosa promoter-binding protein-like 5
At5g59000	miR414	0.77	Zinc finger family protein
At4g31610*	miR414	0.77	REM1 (Reproductive Meristem 1) transcription factor
At5g58980	miR396	0.77	Ceramidase family protein
At5g43730	miR472	0.76	Disease resistance protein
At4g15430	miR855	0.72	Similar to early-responsive to dehydration protein-related
At5g08430	miR414	0.70	SWIB complex BAF60b domain-containing protein/plus-3 domain-containing protein
At2g28510	miR829	0.69	Dof-type zinc finger domain-containing protein
At5g48560	miR778	0.69	Basic helix-loop-helix (bHLH) family protein
At1g27360	miR156	0.68	Squamosa promoter-binding protein-like 11
At3g53310	miR414	0.65	Transcriptional factor B3 family protein
At2g42200*	miR156	0.64	Squamosa promoter-binding protein-like 9
At1g63130	miR400	0.62	Transacting siRNA generating locus
At3g20910	miR169	0.62	CCAAT-binding transcription factor
At2g34960	miR157	0.61	Encodes a member of the cationic amino acid transporter
At1g62670	miR161	0.61	Pentatricopeptide (PPR) repeat-containing protein
At3g57670*	miR854	0.57	Similar to zinc finger

The targets were predicted with the expression dataset of the developmental series. The rate indicates the fraction of runs in which the gene was predicted as a positive in 200 runs. * indicates the gene reported to be involved in the developmental process.

**Table 4:** Top 20 target genes associated with stress responses.

Locus ID	miRNA	Rate	Description
At5g43760	miR854	0.88	A member of the 3-ketoacyl-CoA synthase family involved in the biosynthesis of VLCFA
At5g47250	miR472	0.79	Disease resistance protein
At3g20710	miR859	0.79	F-box/Kelch-repeat protein
At2g36890*	miR847	0.60	Myb-like transcription factor MYB38
At4g28310	miR837-5p	0.60	Unknown protein
At5g41410	miR414	0.55	Homeodomain protein required for ovule identity
At2g25980	miR846	0.53	Jacalin lectin family protein
At5g57590	miR396	0.52	Mutant complemented by E coli Bio A gene encoding 7,8-diaminopelargonic acid aminotransferase
At1g49750	miR854	0.47	Leucine-rich repeat family protein
At5g39710	miR400	0.47	Similar to pentatricopeptide (PPR) repeat-containing protein
At3g13690	miR419	0.47	Protein kinase family protein
At3g18980	miR859	0.45	F-box family protein
At5g43730	miR472	0.45	Disease resistance protein
At2g32760	miR414	0.43	Unknown protein
At1g74840*	miR863-5p	0.43	Myb family transcription factor
At1g80340	miR835-5p	0.42	Encodes a protein with gibberellin 3 β-hydroxylase activity
At1g26210	miR414	0.41	unknown protein
At2g17830	miR859	0.41	F-box family protein
At4g14680	miR395	0.40	ATP sulfurylase
At5g61480	miR870	0.38	Leucine-rich repeat transmembrane protein kinase

The targets were predicted with the expression dataset of stress treatments. The rate indicates the fraction of runs in which the gene was predicted as a positive in 200 runs. * indicates the gene reported to be involved in the stress responses.

#### Developmental-related miRNA targets

AGO7/ZIPPY (At1g69440), a member of the Argonaute family, plays a role in the *TAS3 *ta-siRNA pathway. *TAS3 *ta-siRNAs are required for proper leaf development through the action of AGO7 [32]. SPL5 (At3g15270) and SPL9 (At2g42200) are the members of the SQUAMOSA PROMOTER BINDING PROTEIN-LIKE (SPL) family of transcription factors. Increased expression of *SPL5*, together with two other genes from the same family, *SPL3 *and *SPL4*, promotes vegetative phase change and flowering, and the decreased level of *miR156 *during juvenile-to-adult transition is responsible for this increase [33]. *SPL3 *and *SPL4 *are the validated targets that belong to our training dataset. *SPL9 *is also regulated by *miR156 *and acts redundantly with *SPL15 *in controlling shoot maturation [34]. *AtREM1 *(At4g31610) encodes a protein with features of transcriptional activators and its deduced protein contains three repetitions of a B3-related DNA-binding domain. It may play a role in the organization of reproductive meristems, as well as during flower organ development [35]. *NTT *(*NO TRANSMITTING TRACT *; At3g57670) encodes a C2H2/C2HC zinc finger transcription factor specifically expressed in the transmitting tract. Mutations in *NTT *cause reduced fertility by severely inhibiting pollen-tube movement [36].

#### Stress-related miRNA targets

At1g74840 encodes a protein belonging to the myb family of transcription factors and responds to the CdCl_2 _and NaCl treatments [37]. BIT1 (At2g36890), also a MYB transcription factor, plays an important role in controlling blue light responses [38].

## Discussion

In this study we presented a novel method for screening miRNA targets that are likely to be involved in specific biological processes. Currently, several computational algorithms for miRNA target prediction have been implemented and the majority of them use properties such as the hybridization based on sequence base pairing between miRNA and mRNA or the minimum free energy. Although computational screening has identified a large number of putative miRNA targets, only a small portion of the targets can be validated. In addition, these computational tools do not imply which biological processes might be correlated with the targets. One advantage of our method, by using gene expression profile information, is that it can suggest which target genes have highest priorities to be involved in a specific biological process.

If gene expression profiles of transgenic lines with increased miRNA expression are available, it is possible to do high-throughput and more accurate screening of targets [39]. As the under expressed genes are extracted, putative targets can be defined and the set overlapped with computationally predicted targets can be obtained.

Unfortunately, this kind of high-throughput expression profile dataset is difficult to generate due to the high cost and the labor-intensive experimental process. However, currently many expression profile datasets, which were generated without the context of miRNA are available in the public domains for several organisms. This expression profile information could be a valuable source for miRNA target prediction. Although exclusively using gene expression profiles for prediction does not show very good performance, our results indicate that utilization of expression profiles combined with sequence information can identify condition-specific targets and compensate for the limitations of current sequenced based methods.

We identified miRNA target genes associated with the developmental processes and stress responses at the genomic scale using our proposed method. Our results are supported by previous reports indicating that several genes we identified are involved in the corresponding biological processes. However, the biological functions of most target genes are still largely undetermined. The genes ranked with high priorities in developmental processes or stress responses could be the candidates for further studies in terms of gene regulation. We expect that our application alleviates experimental efforts as it suggests novel candidates with high confidence.

Our method provides a framework for identifying miRNA targets involved in specific conditions. It can be applied to diverse gene expression datasets including cancers, diseases, and other species of which the validated target information is sufficient for training the SVM classifier. Since the free energy for miRNA-target duplex is important to predict the targets in animals, it is possible to combine our method with the method using the minimum free energy of hybridization to improve target prediction and to identify condition-specific targets. Consequently, our approach could contribute to elucidation of gene regulatory programs related to miRNAs and their target genes in diverse biological processes.

## Conclusion

Our results suggested that the gene expression profiles related to specific conditions have the potential to discriminate miRNA targets from non-targets. The combination of gene expression and sequence-based methods ensures retrieval of true targets and targets related to specific biological process. We have shown that in *Arabidopsis *the targets related to the biological processes of developments and stresses were successfully extracted by the proposed method. The same framework can be applied to other biological processes or species.

## Competing interests

The authors declare that they have no competing interests.

## Authors' contributions

JGJ proposed the idea, organized overall procedure, built the dataset for computational experiments and carried out the analysis. ZF developed the idea, provided intellectual guidance and mentorship. All authors read and approved the final manuscript.

## Supplementary Material

Additional file 1 List of validated and putative targets. Click here for file

Additional file 2 Ranked list of features selected by the ranker search method using SVM. Click here for file
